# Job loss-related complicated grief symptoms: A cognitive-behavioral framework

**DOI:** 10.3389/fpsyt.2022.933995

**Published:** 2022-07-22

**Authors:** Janske H. W. Van Eersel, Toon W. Taris, Paul A. Boelen

**Affiliations:** ^1^Department of Clinical Psychology, Utrecht University, Utrecht, Netherlands; ^2^Department of Social, Health and Organizational Psychology, Utrecht University, Utrecht, Netherlands; ^3^ARQ National Psychotrauma Centre, Diemen, Netherlands

**Keywords:** complicated grief, job loss, maladaptive coping, negative cognitions, vignettes

## Abstract

In a significant minority of people, involuntarily job loss can result in symptoms of job loss-related complicated grief (JLCG). The present cognitive-behavioral framework is introduced to explain the underlying processes that may lead to the development and maintenance of JLCG symptoms. Three core processes play a central role, namely (1) negative cognitions related to the job loss and misinterpretation of one's grief reactions; (2) anxious and depressive avoidance strategies to cope with the job loss and its consequences; and (3) insufficient integration of the job loss into the autobiographical memory. These core processes are assumed to interact and reinforce each other, leading to JLCG symptoms. The three core processes can be influenced by certain risk factors, including circumstances surrounding the loss, personality traits, and characteristics of the social environment. JLCG symptoms can lead to additional psychological and practical problems, such as anxiety and depressive symptoms, lower employability, and reduced likelihood of re-employment. This paper explains and illustrates the three core processes with vignettes. Implications of the model for preventive measures and psychological interventions are introduced. It concludes with suggestions for future research on JLCG symptoms.

## Introduction

In 2021, the unemployment rate in the Netherlands was 3.3%, which is equivalent to 306,000 individuals from a population of 17 million (1). Involuntary job loss is considered a major life event that can cause severe emotional distress (2), disruption of identity, social status and relationships (3), and a decrease in physical wellbeing (4). The value of work derives from the importance that society attributes to employment (5). In more individualistic countries where self-actualisation is an important value, one's sense of self is more closely attached to one's work, whereas in more collectivistic countries one's sense of self is often more strongly bound to the group and caring for each other (6). Consequently, the impact of involuntary job loss on one's identity and wellbeing can vary across countries, depending on one's perception of work and job loss. In general, the most common outcome following involuntary job loss is a stable trajectory of healthy mental functioning (7, 8). Only a significant minority develops symptoms of depression, anxiety, and job loss-related complicated grief [JLCG; (3, 9)].

During the last few years research on JLCG symptoms has increased. Factor and latent class analyses have shown that symptoms of JLCG, depression, and anxiety following involuntary job loss represent distinguishable constructs (8, 10, 11). Research has also indicated that JLCG symptoms are associated with low self-esteem, maladaptive coping strategies, and a belief in an unjust world (8, 10, 11). Moreover, scholars have found a relation between JLCG symptoms and identity disruption (3). While these results show that JLCG symptoms are related to increased levels of psychological distress, this does not imply that JLCG should become a novel psychiatric disorder. Notwithstanding this, it remains unclear at present which underlying mechanisms lead to the development and maintenance of JLCG symptoms. It is important to learn more about the characteristics of individuals who develop JLCG symptoms to foster timely identification and treatment of these symptoms and to learn which specific characteristics of JLCG symptoms need to be targeted by these interventions.

From bereavement loss research, it is known that complicated grief requires a different approach in terms of treatment than other forms of bereavement-related emotional distress, such as depression or anxiety (12). A theoretical framework is required to enable us to connect the existing knowledge about JLCG symptoms to possible underlying mechanisms, risk factors, and interventions. Therefore, the aim of this article is to introduce a cognitive-behavioral framework. This may help to provide a deeper understanding of what causes a small, yet significant minority of people to develop JLCG symptoms after involuntary job loss.

First, JLCG symptoms will be described in more detail and illustrated with a vignette. Second, important possible consequences of JLCG symptoms will be discussed. Third, a cognitive-behavioral framework will be introduced based on three core processes, and each core process will be portrayed with a vignette. Fourth, risk factors for the development and maintenance of JLCG symptoms will be considered in relation to this framework. Finally, clinical implications and suggestions for future research directions will be discussed.

## JLCG symptoms

The grief process encompasses a wide array of emotions, cognitions, and behaviors. Part of a healthy grief trajectory are high levels of emotional distress and intense reactions of grief which persist for only a brief period after the job loss, while a person remains capable to function in all aspects of one's daily life (13). In the case of JLCG symptoms this healthy trajectory toward recovery gets disrupted, resulting in grief reactions that persist rather than diminish as time passes by (3, 14). The job loss appears to have shattered fundamental assumptions about oneself and the world (15).

Mirroring the conceptualization of complicated grief after bereavement loss (16), JLCG symptoms include difficulties accepting the changed reality, yearning for the lost job, preoccupation with memories of (circumstances surrounding) the job loss, identity disruption, problems with finding purpose, bitterness and anger, and difficulties with moving on, which, in combination, lead to severe emotional distress and affect functioning in everyday life (14). These JLCG symptoms will be illustrated in the vignette of David.

## Vignette: David^1^

David is 52 years, married and father of two boys. Both he and his wife are currently unemployed and receive social benefits from the government. For over 20 years, he has been a project manager at a metal construction company. He loved this job and devoted a significant amount of time, effort, and energy to it. His customers were satisfied and he was proud of the excellent results he and his team had achieved. That is, until a colleague was promoted to be his executive manager. From David's perspective, things went rapidly downhill from there. He was excluded from meetings and no longer received information essential to carry out his job. He felt overwhelmed and confused. This sequence of events resulted in a labor dispute and an unworkable work environment, which ultimately led to the termination of his contract.

Ten years after this event, David still feels confused, sad, and betrayed. Since then, he has held several jobs, only to lose them again due to his constant preoccupation and yearning for his lost job. He tends to compare every new job with the job he lost, which negatively affects his work performance. He missed the appreciation he used to receive from his clients and colleagues, and he longs for the prestige he gained as an expert in his field. In addition, David frequently gets overwhelmed by memories about his dismissal and the events leading up to it. In particular, the smug look on the face of his colleague when he told him the news, is etched in his memory. He continues to ruminate on those crucial moments, specifically on what he could have done differently to prevent this dreadful end result. Consequently, in each new job he is afraid that history will repeat itself, so he keeps colleagues at bay and finds it hard to trust others. This makes it difficult for him to demonstrate his skills and knowledge and he fails to gain the positive feedback and connection with the team he longs for. Instead, his negative self-image and sense of loneliness are reinforced.

At present, each time David applies for a job, he gets emotional (e.g., sad, or lost for words) when they question him regarding his lost job, and they turn him down. He feels disappointed, confused, and distraught. He finds it tough to keep faith in the future and to find purpose in life. He wants to leave the past behind, but he does not know how.

## Consequences of JLCG symptoms

The vignette of David describes someone who has been suffering from JLCG symptoms since losing his job 10 years ago. As time passes by, JLCG symptoms can result in additional psychological and practical problems creating a downward spiral. For instance, there is preliminary evidence that JLCG symptoms can inflate depressive and anxiety symptoms over a six-month period (17). In general, the emotional distress that follows job loss can lead to cascading psychological and psychosomatic problems (18, 19), stigmatization, social withdrawal (2, 20), physical ailments (21, 22), sleep problems (23, 24), feelings of powerlessness (25, 26), and impaired quality of life (27, 28).

According to the conservation of resources (COR) theory, the depletion of available resources, like financial means, social support, optimism, hope, and self-efficacy, plays an important role in the perceived emotional distress following job loss (29). COR theory states that people are driven to obtain, retain, and protect their valued resources (30). Valued resources in the case of job loss can be time structure, social status, identity, affiliation, and purpose (31). The level of psychological stress caused by the job loss depends on one's appraisal of this loss, the resources that are available to cope with the threat, and the resources (such as time, effort, and skills) invested in the job (32).

Research has shown that individuals who experience mental health issues have a reduced chance of re-employment (33, 34) compared to others. JLCG symptoms can diminish one's employability due to identity disruption (3), loss of self-esteem, increased use of maladaptive coping styles (10, 14), and a decline in social contacts (2). The decrease of available resources and diminishing employability make it more difficult for a person (to continue) to engage in effective job search activities (19). It is conceivable that this potential downward spiral strengthens negative cognitions about one's self, one's life, and one's future, and hinders adaptive coping strategies to deal with the accumulation of problems, making it progressively harder to face the changed reality and the consequences of one's job loss.

## A cognitive-behavioral framework of JLCG

**Figure 1:**
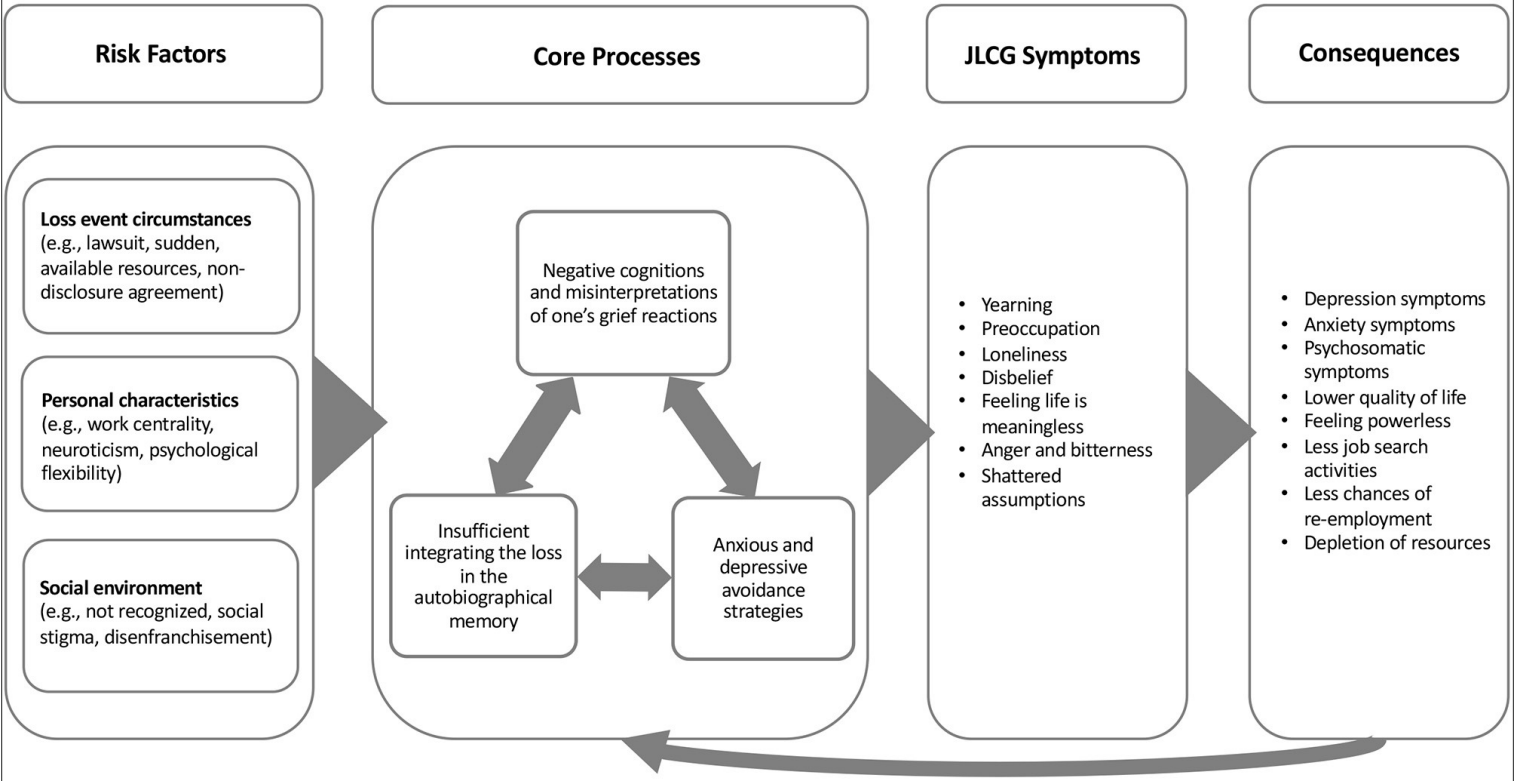
Schematic depiction of the cognitive behavioral framework of job loss-related complicated grief symptoms.

Drawing on a cognitive-behavioral conceptualization of CG following bereavement loss (35), we propose that three processes play an essential role in the development and maintenance of JLCG symptoms. First, negative cognitions about oneself and the world may be reinforced or created as a result of one's job loss, and one's own grief reactions can be misinterpreted as threatening. A second process includes deployment of anxious and depressive avoidance strategies that may help to cope with the loss and its consequences. Third, difficulties with the integration of the loss into the autobiographical memory may block the acceptance of the changed reality. These three processes are critical to the development and maintenance of JLCG symptoms, as schematically depicted in Figure 1. They will be explained in more detail below.

## Negative cognitions and misinterpretations of grief reactions

The first core process is that individuals who suffer from JLCG symptoms hold negative global cognitions related to one's job loss and misinterpret their grief reactions as threatening. These negative cognitions can hinder integration of the loss into the autobiographic memory and can foster the use of maladaptive avoidance coping styles.

According to Janoff-Bulman (15) people hold three abstract fundamental assumptions: “the world is benevolent,” “the world is meaningful,” and “the self is worthy.” In conjunction these assumptions imply that individuals believe that the world is a good place and that they can be optimistic about their own future (36). In addition, people hold specific beliefs about why particular events happen in the world to certain people (37) and have a tendency to believe that one's level of goodness and morality determines one's fate in life [one's “karma” (38)]. In general, most people perceive themselves as good, moral, and capable, relative to others (39). These fundamental assumptions enable people to comprehend the world and themselves. Yet loss and traumatic events can shatter these beliefs. Did they deserve such an event to happen to them? If so, why? Indeed, is the world really “just” after all [cf. (15)]?

Involuntary job loss can shatter one's fundamental beliefs about the world. As a result, job loss and its ensuing consequences can require revision and reconstruction of views around one's self, one's life, one's future, and the world (3). It is conceivable that the intensity of emotional distress following job loss is related to the specific fundamental assumptions that are challenged by the loss. Prior research showed that negative cognitions concerning one self, life, and future (e.g., “My life is meaningless since I lost my job”) were associated with a diminished sense of self and identity confusion (3). Further, researchers have found a negative association between lowered self-esteem and levels of JLCG symptoms (8, 10). A strong global belief in an unjust world was also found to be associated with high levels of JLCG symptoms (10), even when controlling the time elapsed since the job loss (8). Rebuilding one's identity in this new reality, without the lost job, requires revision of fundamental beliefs about one's self, life, future, and the world.

Misinterpretation of one's grief reactions and perceiving them as threatening has been relatively understudied in the case of involuntary job loss. It is conceivable that individuals after the loss of their job may have a tendency to interpret their emotional distress as intolerable, the intensity of their sadness as a sign of losing control, and intrusive thoughts about the loss as a reflection of insanity. Preliminary research showed that these types of cognitions (e.g., “If I really give in to my sorrow, I would go crazy”) were strongly associated with JLCG symptoms (40). It can be postulated that individuals who endorse these types of misinterpretations may experience more intense grief reactions and likely exhibit more avoidance behavior. The first core process is illustrated in the vignette of Carolyn.

## Vignette: Carolyn

Carolyn is single, 51 years old, and has always focused on her career. She worked in public broadcasting for 4 years as a department manager. She excelled in her profession and was highly appreciated by her colleagues and directors. However, when she came back from a three-month sick leave her function was eliminated due to a reorganization.

Before this event 15 months ago, Carolyn was a strong, independent woman, who enjoyed challenging projects and changes. Since the event, she has lost her faith in herself and her future. She feels that the organization has treated her dishonestly and unfairly. Her faith in others has been crushed. For Carolyn her dismissal came out of the blue. No one from the organization informed her on the upcoming changes. Her self-confidence and trust in her own abilities were strongly linked to her work and were shattered by her dismissal. As time passes by, she finds it harder to believe that she is capable of doing anything work-related. She still gets regular calls for job opportunities, which she rejects because she believes she is not ready. She is terrified that if she would get the job, she would not be able to live up to their expectations and will be dismissed again. From the moment she wakes up until she goes to sleep, she ruminates and worries. The prevailing thought on her mind is that she is a worthless person without her lost job.

On top of this, Carolyn is convinced that the way she is reacting to her job loss is abnormal and a sign of weakness. As a result, she is afraid to admit to her environment how she truly feels, denying them the opportunity to correct her misbeliefs. When she is among people, she puts on a brave face and pretends everything is fine. She tries to push forward and suppress her feelings, but the harder she tries, the more intense her feelings and thoughts about her job loss become (e.g., “the world is unfair,” “if people knew how I really feel, they would think I am crazy,” and “I have become worthless”). She feels that she is stuck in a downward spiral and that there is no way out.

## Anxious and depressive avoidance strategies

The second core process is that individuals who experience JLCG symptoms engage in maladaptive coping strategies, obstructing the correction of negative cognitions and misinterpretation of one's grief reactions and blocking the integration of the loss into existing autobiographical memories.

Avoidance behavior is not necessarily problematic and can be a healthy way of coping with loss (41). It is natural that in times of grief the mind oscillates between confronting and avoiding the altered reality (13, 42). This is a way to remain connected to the lost job and to escape from the pain of the permanence of the loss, enabling someone to face the painful reality at one's own pace (41). However, when avoidance strategies dominate the way one deals with the loss, the adaptive learning process of living without the lost job can be hindered, resulting in JLCG symptoms (10, 14). In the current framework, these maladaptive coping strategies are categorized into anxious and depressive avoidance strategies.

### Anxious avoidance strategies

Anxious avoidance strategies can involve avoidance of places (e.g., the company building), people (e.g., former colleagues), and objects (e.g., company documents or memorabilia), which can trigger thoughts or memories about the job loss. For instance, some people are convinced that their emotions must be supressed at all times, because if they even let just a glimpse of their sadness escape, they would be overwhelmed by the emotion. This process is comparable to pressing a ball under water; the harder you push, the more counterpressure is created (43). In order to remain in control of their emotions, they start avoiding more and more places, people, and objects, to avoid (sudden) emotional triggers of their job loss.

Anxious avoidance strategies often represent a form of “experiential avoidance.” This refers to the tendency to suppress, avoid, or disable unwanted internal experiences (e.g., emotions, thoughts, or memories), which paradoxically lead to an increase in frequency and intensity of those unwanted experiences (43). In this vein, it seems conceivable that individuals who view their own grief reactions as threatening, are more likely to fall back on experiential avoidance behavior and encounter more difficulties integrating the job loss and its consequences in the autobiographical memory.

Rather than to avoid the confrontation with the reality of the loss, individuals can also attempt to maintain a strong connection to the lost job (44), for instance, by continuously talking about the circumstances surrounding the loss or dwelling on memories of the period one still had the job. This can be a way to avoid the reality of the job loss, since, as long as someone is focused on the past, one does not have to deal with the reality of the present and the future (41).

### Depressive avoidance strategies

Depressive avoidance strategies can be defined as inactivity and the tendency to withdraw from normal social and recreational activities that were valued before the job loss (35, 45). Individuals can fall back on this behavior when, for instance, psychosocial or financial resources to act differently are lacking (32, 46).

Negative expectations also seem to play a crucial role in driving depressive avoidance strategies. Individuals who expect to be rejected by their social environment are more likely to engage in social withdrawal (47). The same applies to individuals holding negative expectations about the effect of, and their ability to participate in potentially helpful social or recreational activities (35). These types of negative cognitions may lead to depressive avoidance behavior which, in turn, prevents the correction of these negative cognitions.

Depressive avoidance strategies are likely to contribute to the development and maintenance of JLCG symptoms by enhancing feelings of yearning, a sense of meaninglessness, and preoccupation with the job loss. Social withdrawal, inactivity, and apathy can hinder correction of one's global negative cognitions about one self, life and the future and, as a result, make it more difficult to move on and set new goals (40). For instance, feelings of shame and self-blame regarding one's job loss and negative cognitions about one self (e.g., “I am worthless, since I lost my job”) are likely to intensify social withdrawal, out of fear what others might think. Depressive avoidance strategies can also lead to a reduction of perceived social support and weaken the strength of one's social network, making it harder to obtain new employment (48). The vignette of John illustrates the role of anxious and depressive avoidance in JLCG.

## Vignette: John

John is married, 60 years old and has three adult children. He worked as teacher at an elementary school for 15 years. He saw this as his calling, in particular working with children who need some extra attention to flourish. He invested much time and energy in his job and he often stayed late or worked from home to prepare the lessons for the following day. He did this with pleasure and saw it as part of his job as a teacher.

After various reorganizations and a fatal accident on school premises, the culture at the school started to change. According to John, the school started to feel unsafe and distrust among colleagues and with the principal ensued. John began to suffer from stress and anxiety symptoms. Communication with the school principal became tense, resulting in a labor conflict. The organization offered John money to terminate his contract in exchange for a non-disclosure agreement on everything that had occurred. John declined, which resulted in a lawsuit that lasted for 1.5 years. Losing his job became inevitable; this was 3.5 years ago.

At present, John avoids going to the school premises and other places where he might meet his former principal or colleagues. This is challenging, since the school is located in a town nearby, where he used to buy the weekly groceries. At first, his wife took over this task, because driving a car became difficult for him due to concentration problems following his job loss and also due to his fear of running into former co-workers or children's parents. Last year he started to drive again, which is going well as long as he stays away from any potential emotional triggers related to his job loss.

Till this day, John still avoids working with children. Although he was excellent at his job and children as well as their parents spoke highly of him, he is afraid it could trigger memories of his job, his dismissal, and associated intense emotions. He ruminates daily on the events surrounding his job loss and the lawsuit, in which the sense of injustice prevails despite the lawsuit victory. He still misses the relationships with his former colleagues and the sense of purpose that came through working together on a shared goal. His negative thoughts and avoidance behavior bind him to the past, making it hard for him to let go and move on.

## Loss integration in autobiographical memory

The autobiographical database contains general information on events that have occurred in complex knowledge structures, lying at the basis of memories and schemata about one's life (49). In the case of a healthy trajectory after job loss, loss-related information (e.g., feelings, thoughts, memories) is gradually linked to the existing knowledge which enables the changed reality, without the lost job, to sink in (35). This integration process is essential to slowly reducing high levels of emotional distress and intrusive memories.

In the case of complicated grief, the information on the permanence of the loss is insufficiently integrated into the autobiographical memory (50). As a result, the loss event can easily trigger loss-related thoughts, feelings, and emotions in the associative network of memory (35). Due to this insufficient integration in the autobiographical memory, individuals continue to feel stunned and shocked. They seem incapable of comprehending how and why this has happened to them. Often, job loss is a result of human decisions that can make it more difficult to regard the loss as permanent. As a result, it may be harder for a person to accept the irreversibility of the job loss and integrate the loss into the autobiographical memory; for instance, it might be tempting to believe that there is still a way to undo the job loss by filing a lawsuit. Preliminary results showed that people who experience JLCG symptoms find it difficult to accept that the job loss is irreversible, feel overwhelmed by memories of the job loss, and are preoccupied with the job loss (3, 11). They experience disbelief concerning the permanence of their lost job and keep playing the sequences of events over and over in their head, trying to figure out where it went wrong [e.g., (10, 51)]. The third core process of insufficient integration of the job loss into the autobiographical memory is illustrated in the vignette of Lisa.

## Vignette: Lisa

Lisa is 43 years old, married, and has no children. She worked for a global software company for 8 years as a learning and development specialist. She loved the diversity of her job, the complexity of the projects, the traveling around the world, but above all the appreciation she received from her executive and colleagues. The company was growing rapidly and reorganizations were the order of the day. Deep down, Lisa knew that 1 day her job would be outsourced to another country. Then 1 day, indeed, she heard she had lost her job. Nevertheless, she was shocked by this news and could not believe that this was happing to her.

Eighteen months have passed since the news broke. To this day, almost every night Lisa dreams about the projects she completed, the meetings with her team, and the problems she solved for clients. When she wakes up, she feels vivid and enthusiastic, until after a few moments, reality sets in and feelings of sadness and confusion overwhelm her. It takes her a while to pull herself together before she can begin her morning routine. Although Lisa quickly found a new job as a consultant at an employment agency, she still longs for her previous job, particularly the sense of competence and appreciation she gained in this job. Even during her current work, her mind wanders to memories of her lost job, and she has to remind herself that she no longer works there. Although she likes her colleagues, clients, and tasks in her current job, she feels disconnected and estranged. Lisa believes she has lost a part of herself and she first needs to figure out who she is before she will be able to move on. Therefore, against all odds, she keeps waiting and hoping that 1 day the phone will ring and she can return to her old position.

## Risk factors

As schematically depicted in Figure 1, within the framework it is proposed that the three core processes can be influenced by specific risk factors, including circumstances surrounding the job loss, personality traits, and characteristics of the social environment. From each category several important examples will be discussed. This list, however, is not exclusive and can be extended. This presumed effect of these variables will be illustrated with examples for each category of risk factors.

### Circumstances surrounding the loss event

When someone is involved in a *labor conflict* or *lawsuit*, emotions such as anger and bitterness, as well as the negative cognitions about the job loss, can serve a purpose, namely to keep pursuing justice. These types of conditions can create a perfect grounding to amplify and strengthen negative cognitions related to one's job loss.

Experiencing one's job loss as *sudden* or *unexpected* (11) and not being able to say *farewell* in an appropriate way after the job loss (14), have also been related to JLCG symptoms. It is conceivable that this can make it more difficult for someone to accept the irreversibility of the job loss.

Furthermore, an employer may insist that the employee signs a *non-disclosure agreement* to end their labor contract. Under these circumstances, the employee is not allowed to talk to anyone about the events that have led to one's job loss. As a result, this person is unable to discuss their negative cognitions with their social environment, hindering the correction of these negative cognitions.

Finally, from the perspective of the COR theory, the range of *available resources* and *loss of resources* (e.g., income, social contacts, status, and self-efficacy) can influence the intensity of distress surrounding the job loss (29). For instance, perceived *financial strain* has been associated with an increase of avoidance behavior (10), whereas higher *unemployment benefits* have been found to be related to better psychological wellbeing and less financial strain (22, 52). Hence, it seems plausible that higher social benefits may have a mitigating effect on the engagement in anxious and depressive avoidance strategies.

### Personality traits

Certain personality traits are likely to influence the three core processes. For instance, *work centrality* refers to the extent to which work is important within one's life (53). A high degree of work centrality implies that an individual strongly *identifies* with one's job (3), which seems especially common in individualistically-oriented countries. As a result of this identification, it is harder to integrate one's job loss with one's existing memories and image of oneself.

In addition, *neuroticism*- the tendency to view the world and the self negatively and to experience intense negative emotions in response to stress (54) – can render someone susceptible to negative cognitions following job loss.

Similarly, a limited degree of *psychological flexibility* – one's ability to cope, accept, and adjust to difficult situations - is related to an increased tendency toward experiential avoidance (43). This may lead to a more frequent reliance on anxious avoidance coping strategies after involuntary job loss.

### Reactions from the social environment

Reactions of the social environment may also affect the three core processes. When the impact of one's job loss is *not recognized* or *supported* by a person's social environment, it can increase feelings of shame and guilt (55). Negative reactions from one's social environment and *disenfranchisement* from grief can interfere with the integration of the loss into the current concept of self.

Job loss is still often associated with a *social stigma* and internalized feelings of *blame* (20), which can increase depressive avoidance behavior. An empathic social environment can play an important role by encouraging someone to go out, become more active, and undertake activities. Nevertheless, the *availability* of one's social environment may decrease as a result of one's job loss and interactions with family and peers may change (2), leading to further social isolation (56), and increased depressive avoidance behavior.

## Implications of the cognitive-behavioral framework of JLCG symptoms

To alleviate JLCG symptoms, interventions could be used to target the three core processes of this cognitive-behavioral framework. That is, interventions could focus on (1) identifying and altering problematic cognitions related to the job loss and misinterpretations of one's grief reactions, (2) replacing anxious and depressive avoidance strategies with more adaptive strategies to cope with the job loss, and (3) stimulating integration of the job loss and its consequences into one's autobiographical memory.

Since research on JLCG symptoms is relatively new, no studies have yet been conducted on the effectiveness of treating these symptoms. Nonetheless, cognitive behavioral therapy (CBT) has been found to be effective in the alleviation of complicated grief symptoms following bereavement loss (57, 58). It seems conceivable CBT interventions may also help mitigating JLCG symptoms. Another promising candidate to reduce JLCG symptoms is acceptance and commitment therapy (ACT), which has been found to be effective in treatment of mental problems (59), including complicated grief (60). ACT is based on a contextual form of behavior analysis and emphasizes the functions of problematic behaviors (43). One key goal of ACT is to achieve increased psychological flexibility (61). Suggestions for preventive measures, psycho-education, and psychological interventions based on CBT and ACT will be provided in the succeeding sections.

### Preventive measures

Several pointers for preventive measures and treatment options can be derived from the cognitive-behavioral framework. As noted, preliminary evidence shows that perceiving the job loss as unfair, sudden and beyond one's control can influence the three core processes (8, 11, 17, 62). These types of circumstances of the job loss can be influenced by the employer up to a certain level. For instance, involving the employee early on in the process and straightforward *communication* will diminish the chance that the employee will perceive their job loss as unexpected. An *exit interview* after one's dismissal can be a useful instrument as well (63). This conversation between the employer and the departing employee can offer the opportunity to discuss pending questions, review achievements, share mutual appreciations, and give constructive feedback. Open communication, sincere interest, respect, and authenticity are key factors for a constructive exit interview (64). On the one hand, this will increase the chance that the employee feels seen and acknowledged in their loss. On the other hand, an exit interview can correct some negative assumptions about the cause of the dismissal and reduce the risk that the employee considers the dismissal unfair. Finally, discussing the options for saying *farewell* to the company, colleagues, customers, and how to structure the last period to transfer all the work in a proper manner, will give the employee a greater sense of control during a time with many uncertainties.

### Psycho-education

When a person with JLCG symptoms, i.e., the client, seeks help, the therapist should provide *psycho-education* and *normalize* the JLCG symptoms as a preventive measure, explaining to the client that the experienced JLCG symptoms are understandable reactions following one's job loss. This will decrease the chance that clients will negatively misinterpret their grief symptoms as abnormal and threatening. It may help them to better understand what is going on and the process that they are going through, namely, that reactions like yearning, preoccupation with the loss, feeling numb, anger, and a sense of futility, are all part of the grieving process. Therefore, it is advisable to explain the three core processes to clients, also addressing how they can amplify each other. This perspective can help them to see that one's negative thoughts and feelings may be a consequence of the grief and can fuel JLCG symptoms. For instance, when clients believe to be worthless without their lost job, it can be harder to connect with other people (“they will think I am worthless too”) and to undertake activities (“I am not good for anything”). Discussing examples from one's daily life, in particular specific thoughts, cognitions, and behaviors that are most upsetting to clients, will help them to understand how their behavior pattern can interfere with their process of adjustment.

### Psychological interventions

For each of the three core processes, we offer examples of psychological interventions from CBT and ACT. For the first core process, the key is to modify *negative cognitions* and *misinterpretations of grief reactions*. Effective CBT interventions to achieve this are c*ognitive restructuring* techniques (65). These techniques can help clients to identify and examine the utility and validity of their cognitions and beliefs. For instance, when clients believe the future holds no meaning since the job loss, categorizing a list of evidence in favor of as well as opposed to this statement can be helpful, to subsequently seek for alternative theories based on the given evidence, to the modify this negative belief into a more manageable form (66).

When clients strongly identify with limiting thoughts, cognitions, feelings, or identity labels (e.g., “If I really give in to my sorrow, I would go crazy”) ACT interventions such as *defusion* techniques can aid them to handle these unwanted thoughts and feelings and increase one's psychological flexibility. Defusion is basically the deliteration of language in order to strip words and sentences of their symbolic meaning (43). This can be achieved by repeating a simple word or sentence, like “I am weak,” for several minutes with a voice modifier, until the sentence starts losing its symbolic meaning. The goal of this exercise is to expand the associative network surrounding the cognition “I am weak” with alternative thoughts and feelings to reduce the level of emotional distress (61).

For the second core process, the central aim is reducing the utilization of *anxious and depressive avoidance strategies*. From a CBT perspective, when clients are avoiding reminders of the job loss (e.g., places, people, or objects) *exposure* procedures can be deployed to correct misinterpretation of one's grief reactions and to diminish avoidance attempts (35). Examples of this are setting up a concrete plan and stimulating clients to reach out to former colleagues, or by asking them to bring documents or photo's which are related to the lost job to discuss what they represent to them.

*Behavioral activation* strategies from CBT can be implemented to break the negative circle of depressive avoidance strategies and the apathy and withdrawal that follows. The aim of these strategies is to structure attempts to undertake activities which can improve one's mood and quality of life (67). If these activities, in addition to being pleasant and meaningful, are also linked to activities that clients enjoyed before the job loss, this can also facilitate the reconstruction of one's self (3).

From the perspective of the ACT, *acceptance* is a process of unconditionally allowing and actively inviting all thoughts, feelings, experiences in the client's life that they would prefer to suppress or avoid and that cannot be influenced by one's behavior (68). Hence, clients need to learn to distinguish between *pain*, which is a part of life and caused by direct circumstances, and *suffering*, which has no clear cause and arises from resisting pain (43). The goal is to develop willingness in the client to accept psychological consequences of life events, such as one's job loss, as they come rather than trying to avoid emotional triggers of the job loss by employing anxious avoidance strategies.

To address depressive avoidance behavior, ACT interventions focused on *exploring personal values* may be effective (43). For instance, if clients categorize justice, friendship, and clarity as their main personal values, the next step would be to support them to define what a value-oriented life would precisely entail; particularly, which steps and actions can be taken to increase their sense of justice, friendship, and clarity in their life. The answers can help to draw up a specific action plan to encourage clients to undertake small steps toward committed action and a value-oriented life (61).

With respect to the third core process, facilitating *integration in the autobiographical memory* of the job loss and its consequences, a key aim is to help clients to rearrange their internal and external world in such way that the loss is taken into account. From a CBT perspective, this can be achieved by discussing the clients' narrative of the job loss. The next step is to carefully review memories of the circumstances leading up to the dismissal, zooming in on the most painful and meaningful elements (65). Facing these painful moments, feelings, and thoughts instead of avoiding them can help the client to integrate the job loss with their existing memories and image of themselves.

From the perspective of the ACT, interventions focused on the *self* can help to integrate the job loss into the client's autobiographical memory. Clients will unravel the self on three levels, namely the self as content, as process, and as context, and practice with the ability to take different perspectives. Mapping aspects of the self on different moments in time, can help clients to get in touch with that part of the self which is constant throughout time, and will remain the same even without the lost job (68).

## Future research

One of the goals of the framework presented here is to stimulate systematic research. We have formulated several areas of interest for future research on JLCG symptoms.

### Areas for further research

From research by Bonanno et al. (7) an estimation can be made that JLCG symptoms occur in ~18% of the cases of involuntarily job loss. However, this research focused on emotional distress and quality of life, rather than on JLCG symptoms. Before we can determine the prevalence of JLCG symptoms, we first need to conduct more systematic research whether JLCG symptoms substantially differ from *uncomplicated/healthy grief* , *depression*, and *anxiety* symptoms after involuntary job loss.

Job loss often leads to a decrease of available resources and over time depletion of resources [e.g., money, time, social contacts, “favors” (32)]. The degree to which available resources and the depletion of resources are involved in the development and maintenance of JLCG symptoms needs to be thoroughly examined. This knowledge could provide more guidance for tailor-made preventive measures and early interventions.

The influence of negative, but also positive cognitions on JLCG symptoms needs to be unraveled to identify vulnerable individuals after involuntarily job loss and to create customized psychological interventions. It seems conceivable that these interventions, for instance from CBT or ACT, should focus on modifying the negative cognitions and biases created by the job loss and reinforce positive cognitions, such as hope, optimism, and self-efficacy, to reduce the JLCG symptoms.

Finally, possible differences across countries regarding the development and maintenance of JLCG symptoms are currently unknown, while the meanings of work and job loss are likely to vary among cultures. For instance, it is conceivable that workers in more individualistic oriented countries may be at greater risk of developing JLCG symptoms than those in more collectivistic oriented countries due to disruption of one's identity.

## Data availability statement

The original contributions presented in the study are included in the article/supplementary material, further inquiries can be directed to the corresponding author.

## Ethics statement

Written informed consent was obtained from the participants for the publication of any potentially identifiable data included in this article. Ethics review and approval/written informed consent was not required as per local legislation and institutional requirements.

## Author contributions

JE, TT, and PB: writing-review and editing. JE: writing-original draft. PB and TT: supervision. All authors contributed to the article and approved the submitted version.

## Conflict of interest

The authors declare that the research was conducted in the absence of any commercial or financial relationships that could be construed as a potential conflict of interest.

## Publisher's note

All claims expressed in this article are solely those of the authors and do not necessarily represent those of their affiliated organizations, or those of the publisher, the editors and the reviewers. Any product that may be evaluated in this article, or claim that may be made by its manufacturer, is not guaranteed or endorsed by the publisher.

## Footnotes

^1^All vignettes described in the article are based on real cases. Names and personal details have been modified to ensure anonymity of the participant. Written informed consent was obtained from the participants for the publication of any potentially identifiable data included in this article.
